# A Rare Case of Other Iatrogenic Immunodeficiency-Associated Lymphoproliferative Disorders With Multiple Oral Mucosal Lesions During Methotrexate Therapy

**DOI:** 10.7759/cureus.58995

**Published:** 2024-04-25

**Authors:** Itsuki Hayashi, Makoto Toida

**Affiliations:** 1 Department of Oral and Maxillofacial Surgery, Sugita Genpaku Memorial Obama Public Hospital, Obama, JPN

**Keywords:** oral mucosa, tongue, multiple lesions, methotrexate, lymphoproliferative disorders

## Abstract

As of the most recent WHO classification of immunodeficiency diseases, lymphoproliferative disorders that occur during treatment with immunosuppressive drugs are classified as "other iatrogenic immunodeficiency-associated lymphoproliferative disorders (OIIA-LPDs)" other than post-transplant lymphoproliferative disorders. Most patients with OIIA-LPD have rheumatoid arthritis as the underlying disease. Research indicates that approximately half of people diagnosed with OIIA-LPD see a remission of their lesion after stopping treatment with methotrexate (MTX), a drug used in rheumatoid arthritis treatment. Hereby, we present the case of an 81-year-old woman with rheumatoid arthritis who developed OIIA-LPD at the bilateral lingual margins. The patient had been receiving MTX for the preceding 10 years. After determining that OIIA-LPD was MTX-related, the patient underwent MTX withdrawal and was treated conservatively. The lesion resolved one month after MTX withdrawal. This case report confirms immunosuppressive drug withdrawal as a potentially effective treatment for multiple OIIA-LPDs of the oral mucosa.

## Introduction

Other iatrogenic immunodeficiency-associated lymphoproliferative disorder (OIIA-LPD) is a lymphoproliferative disease occurring in patients receiving immunosuppressive drugs for rheumatoid arthritis [1].

The pathogenesis of OIIA-LPD is influenced by autoimmune disease activity, Epstein-Barr virus infection, immunosuppressive medications, and aging [2]. Clinical manifestations of OIIA-LPD are often fever, general malaise, weight loss, and superficial lymphadenopathy [2-3]. Among OIIA-LPDs, half of the lymphomas occur in the lymph nodes and the other half at extranodal sites [3]. Moreover, lymph node lesions are mainly lymphadenopathy, whereas extranodal lesions may form ulcers or masses in the gastrointestinal tract, skin, soft tissues, and oral mucosa [3].

The first treatment after OIIA-LPD development is watchful waiting following the cessation of immunosuppressive drugs [2]. Additional therapy is not required for patients without a relapse or regrowth event, but chemotherapy is administered when a relapse or regrowth event develops [2]. 

In this study, we report a rare case of OIIA-LPD with multiple lesions in the oral mucosa.

## Case presentation

An 81-year-old woman presented with a painful ulcer on the bilateral border of the tongue that had persisted for three weeks. She had a history of rheumatoid arthritis and had been receiving methotrexate (MTX) treatment (6 mg, once weekly) for 10 years.

**Figure 1:**
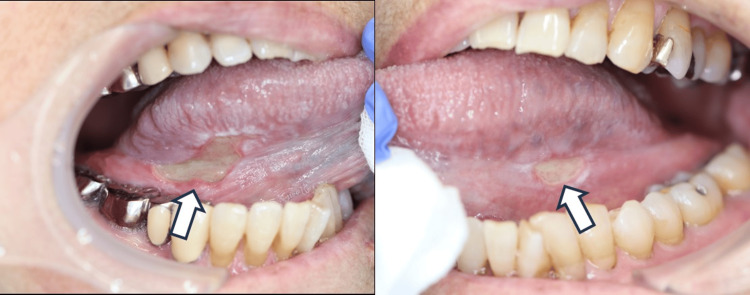
Image of ulcers on the bilateral border of the tongue at the time of initial examination (arrow)

At the time of the patient's first visit to the department, her blood tests revealed a white blood cell count of 8,200/µL and a sIL-2 receptor level of 521 U/ml. She denied any smoking or drinking habits. Her physical examination revealed no regional or generalized lymphadenopathy. Upon her intraoral examination, we observed two well-defined, deep-seated ulcers, each 1.5-2 cm in size, on the bilateral borders of the tongue. Contact pain was present with no palpable induration around the ulcer (Figure 1).

**Figure 2:**
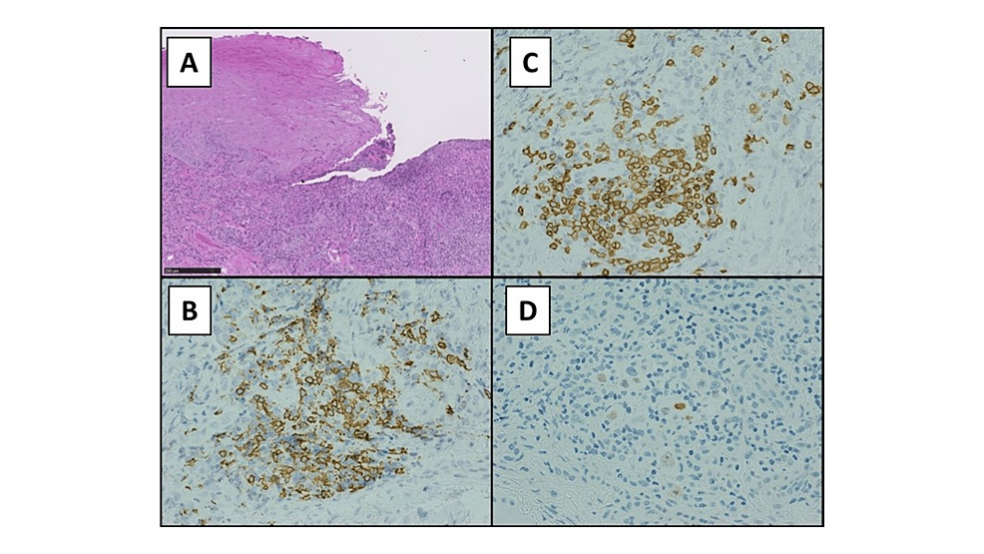
Histopathological examination image of the lesion (A): low magnification of an ulcer on the tongue reveals a highly cellular lymphocytic infiltrate deep into the submucosal layer (hematoxylin and eosin (staining, scale bar = 500 µm); (B-C): the cells are B-cell in origin, being immunoreactive to CD20 (as seen in B; brown signals) and to CD30 (as seen in C; brown signals); (D): the cells are positive for Epstein-Barr virus-encoded early RNA via in situ hybridization (brown signals; EBER staining). RNA: ribonucleic acid; EBER: Epstein-Barr virus encoding region

The differential diagnoses included lymphoproliferative disease and malignancy, as the lesions were refractory and the patient displayed a history of long-term immunosuppressive treatment. We performed an incisional biopsy of the tongue lesions and detected atypical lymphocytes infiltrating the ulcer base, characterized by positive staining for CD20 and CD30 as well as for the Epstein-Barr-encoding region upon in situ hybridization. Based on the MTX treatment history and the clinical and histopathological features, we diagnosed the patient with OIIA-LPD (Figures 2A-2D).

**Figure 3:**
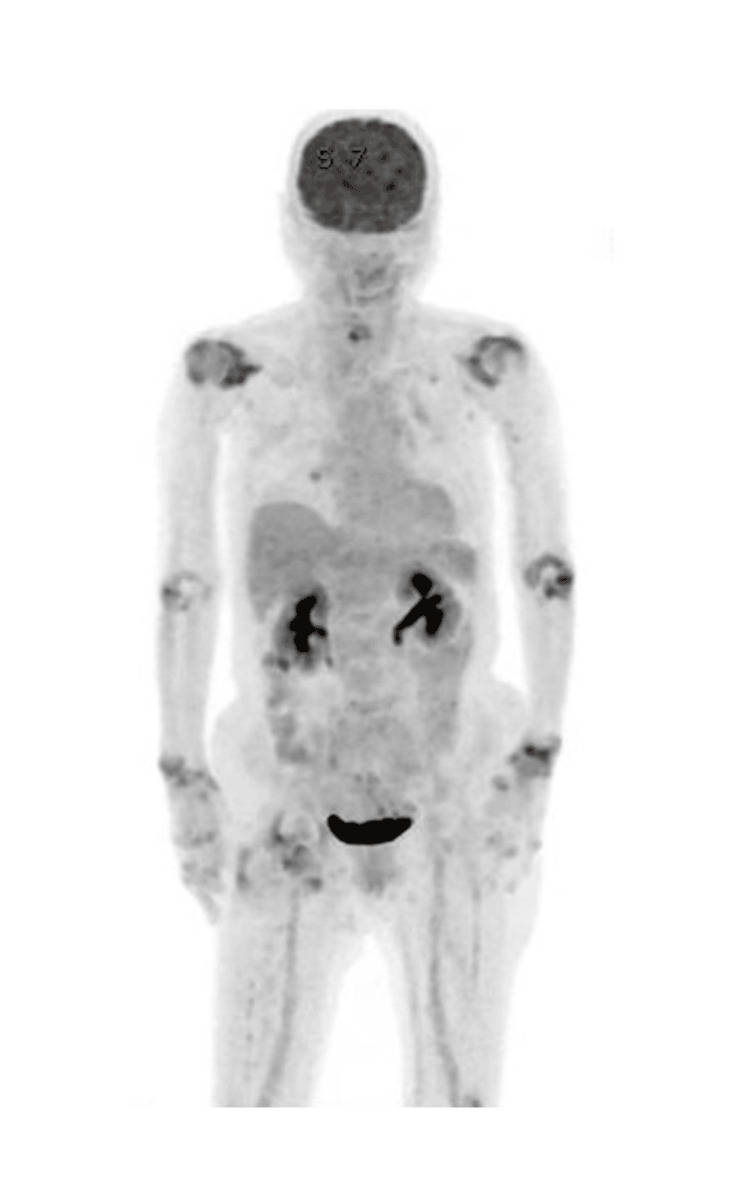
The PET/CT image of the whole body Fluorodeoxyglucose (FDG) uptake is shown in joints with rheumatoid arthritis. No evidence of FDG uptake is seen in lymph nodes or organs throughout the body.

We performed positron emission tomography-computed tomography (PET-CT) to differentiate systemic lymphoma, indicating no multi-organ involvement (Figure 3).

**Figure 4:**
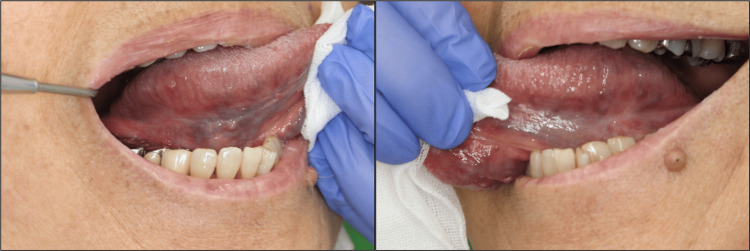
Image of an ulcer on the bilateral border of the tongue two years and five months after discontinuation of methotrexate

As the rheumatoid arthritis was well controlled, MTX was discontinued, and the patient's treatment was changed to prednisolone monotherapy (4 mg/day). The tongue lesions healed one month after the MTX discontinuation. Two years and five months after discontinuation of MTX, there was no recurrence of the lesions (Figure 4).

## Discussion

The WHO divides immunodeficiency-associated lymphoproliferative diseases into four categories: lymphoproliferative disorders with primary immune disorders, lymphomas associated with human immunodeficiency virus infection, post-transplant lymphoproliferative diseases, and OIIA-LPD [4].

Although rheumatoid arthritis, Epstein-Barr virus infection, and therapeutic agent (e.g., MTX)-related immunosuppressive effect-associated immunological abnormalities suggestibly cause LPD, the conclusion remains unclear on the subject [4].

However, since certain LPD cases could be cured by immunosuppressive drug (including MTX) discontinuation, the immunosuppressive effect of drugs was considered the potential cause of LPD onset [4]. Although approximately 40%-60% of OIIA-LPD cases are treated by immunosuppressive agent discontinuation, the cure rate varies depending on the LPD subtype [5-7].

The OIIA-LPD treatment comprises immunosuppressive drug discontinuation (followed by tumor regression within one to two weeks) and a two-week follow-up upon definitive diagnosis [8]. If the tumor exhibits a regressive trend, the patient should be followed up. In principle, immunosuppressive agent re-administration should be avoided even when remission is achieved successfully [8]. If remission cannot be achieved or the tumor relapses after remission, the treatment should be similar to that for malignant lymphoma [8].

In the hereby presented case, the lesion shrank approximately one week after MTX discontinuation, and multiple lesions healed in the oral mucosa after one month. Methotrexate was not resumed even when the LPD was cured [2].

Concerning the OIIA-LPD prognosis, the five-year survival rate is reportedly 58.9%, which is worse than that of general malignant lymphoma (74.6%) [9, 10]. Various factors suggestibly contribute to poor OIIA-LPD prognosis, including long-term immunosuppression and the existence of cases with delayed therapeutic intervention timing due to follow-up intervention upon treatment discontinuation [9].

Immunosuppressive drug discontinuation, including that of MTX, exacerbates rheumatoid arthritis symptoms, results in poor rheumatoid arthritis control, and increases the risk of malignant lymphoma development [8]. Upon immunosuppressive drug discontinuation, both LPD and rheumatoid arthritis states should be carefully monitored.

In the present case, LPD did not recur until the last follow-up, two years and five months after MTX discontinuation. Moreover, rheumatoid arthritis was well controlled with prednisolone monotherapy. Compared to previous cases, early discontinuation of MTX reduced the progression of lesions. Regular dental visits may be useful for the early detection and prevention of lesions in patients taking oral immunosuppressive drugs. This result suggests that MTX discontinuation might be useful in treating OIIA-LPD with multiple lesions in the oral mucosa.

## Conclusions

The development of OIIA-LPD is associated with immunosuppressive drugs, including MTX administration. The discontinuation of the causative agent might thus be helpful. Hereby, we presented a case that suggested that MTX discontinuation could cure OIIA-LPD with multiple lesions of the oral mucosa.
